# Molecular Mechanisms Associated with Brain Metastases in HER2-Positive and Triple Negative Breast Cancers

**DOI:** 10.3390/cancers13164137

**Published:** 2021-08-17

**Authors:** Sarah Bryan, Isabell Witzel, Kerstin Borgmann, Leticia Oliveira-Ferrer

**Affiliations:** 1 Department of Gynaecology, University Medical Center Hamburg-Eppendorf, 20246 Hamburg, Germany; sarah.bryan@stud.uke.uni-hamburg.de (S.B.); iwitzel@uke.de (I.W.); 2 Center of Oncology, Laboratory of Radiobiology & Experimental Radiooncology, Department of Radiotherapy and Radiooncology, University Medical Center Hamburg-Eppendorf, 20251 Hamburg, Germany; borgmann@uke.de

**Keywords:** breast cancer, breast cancer subtype, breast cancer brain metastasis, mechanisms of metastasis

## Abstract

**Simple Summary:**

Breast cancer, the most common malignant tumor among women worldwide, remains an incurable disease once it has spread to the brain. Past research has shown that a primary breast cancer’s biology is an important determining factor predisposing its ability to form brain metastases. This review summarizes our current understanding of which genes, mutations, and molecules cause this increased ability to spread to and survive in the brain, specifically focusing on the different stages of this process. This knowledge may help us develop more effective, tumor-specific therapies and, as such, increase the chance of recovery for patients with breast cancer brain metastases.

**Abstract:**

Breast cancer (BC) is the most frequent cause of cancer-associated death for women worldwide, with deaths commonly resulting from metastatic spread to distant organs. Approximately 30% of metastatic BC patients develop brain metastases (BM), a currently incurable diagnosis. The influence of BC molecular subtype and gene expression on breast cancer brain metastasis (BCBM) development and patient prognosis is undeniable and is, therefore, an important focus point in the attempt to combat the disease. The HER2-positive and triple-negative molecular subtypes are associated with an increased risk of developing BCBM. Several genetic and molecular mechanisms linked to HER2-positive and triple-negative BC breast cancers appear to influence BCBM formation on several levels, including increased development of circulating tumor cells (CTCs), enhanced epithelial-mesenchymal transition (EMT), and migration of primary BC cells to the brain and/or through superior local invasiveness aided by cancer stem-like cells (CSCs). These specific BC characteristics, together with the ensuing developments at a clinical level, are presented in this review article, drawing a connection between research findings and related therapeutic strategies aimed at preventing BCBM formation and/or progression. Furthermore, we briefly address the critical limitations in our current understanding of this complex topic, highlighting potential focal points for future research.

## 1. Introduction

Breast cancer (BC) is the most frequently diagnosed malignancy and most frequent cause of cancer-associated death for women worldwide [1]. As the diagnosis and treatment of primary BC improves, the importance of understanding, foreseeing, and combating the development of metastatic BC increases. Up to 90% of deaths associated with the disease are cases of metastatic BC, most commonly to the lung, liver, brain, and bone [2].

According to current literature, BCs can be classified into up to ten molecular subtypes [3]. However, only four of these are currently clinically relevant and can be determined by immunohistochemistry and are therefore the focus of this review: luminal A, luminal B, human epidermal growth factor receptor-2-enriched (HER2-positive), and triple-negative (Table 1) [3,4]. Subtype-assignment, as first proposed by Perou and Sorlie in 2000, is in part based on expression levels of estrogen and progesterone hormone receptors (ER and PR respectively) and HER2 [5,6]. These factors can either be expressed (positive) or not expressed (negative) on the tumor surface. Tumors lacking these three receptors are regarded as triple-negative BC (TNBC; ER-, PR-, HER2-). A further immunohistochemical marker, Ki67, has henceforth gained importance as an indicator of tumor-proliferation, especially aiding in the differentiation between subtypes luminal A and B [7,8].

Bone metastasis is the most common metastatic site in BC (~67%), followed by liver and lung (~40% and ~37%, respectively). Metastasis to the brain occurs in 10–30% of metastatic BC patients; however, it represents the most intractable issue for patients and a significant and growing hurdle for clinicians [12,13]. The biological mechanisms which promote and hinder brain metastases (BM) formation remain largely unknown, as do the intricacies of pharmacologically overcoming the blood-brain barrier (BBB). Thus, we are yet to develop an effective treatment against breast cancer brain metastases (BCBM). This review will present and evaluate some of the recent insights into the influence of BC molecular subtype on BCBM formation and discuss how these new findings may help to combat BCBM in the future.

A primary BC’s subtype is a highly important determinant of prognosis and patient survival, not only because receptor expression determines the effectiveness of certain therapies but also because a strong association between subtype and metastatic pattern can be observed. Focusing on BM, it is well documented and widely accepted that the HER2-positive and TNBC subtypes are associated with an increased risk of developing BCBM [9,10,13,14,15,16,17,18,19]. In primary breast cancer, the overall 5-year cumulative incidence of BM is 1.7%, 0.1% for luminal A, 3.3% for luminal B, 3.7% for HER2-positive, and 7.4% for TNBC [11] (Table 1). Thirty–fifty% of metastatic HER2-positive patients and 25–46% of metastatic TNBC patients develop BM [17,20]. Interestingly, HER2-positive and TNBC tumors together are responsible for nearly one-quarter of all newly diagnosed BC metastases [21]. Furthermore, the median time between primary BC diagnosis and BCBM development, as well as the median survival post-BCBM-diagnosis, is shortest for patients with TNBC and HER2-positive tumors [20,22,23].

These observations, and the logical questions they evoke, form the basis of this review article. How does a tumor cell’s molecular subtype influence its ability to separate from the primary tumor, circulate within the body, cross the BBB and invade the brain? Which molecular mechanisms explain the propensity of HER2-positive and TNBC to metastasize to the brain? Can the molecular subtype of a primary tumor reliably predict metastatic propensity to the brain? Moreover, finally, how can we use this knowledge to develop specific therapies to combat BCBM?

## 2. Stages of Brain Metastasis Development: Key Factors Associated with HER2-Positive and Triple Negative BC Subtypes

**Figure 1:**
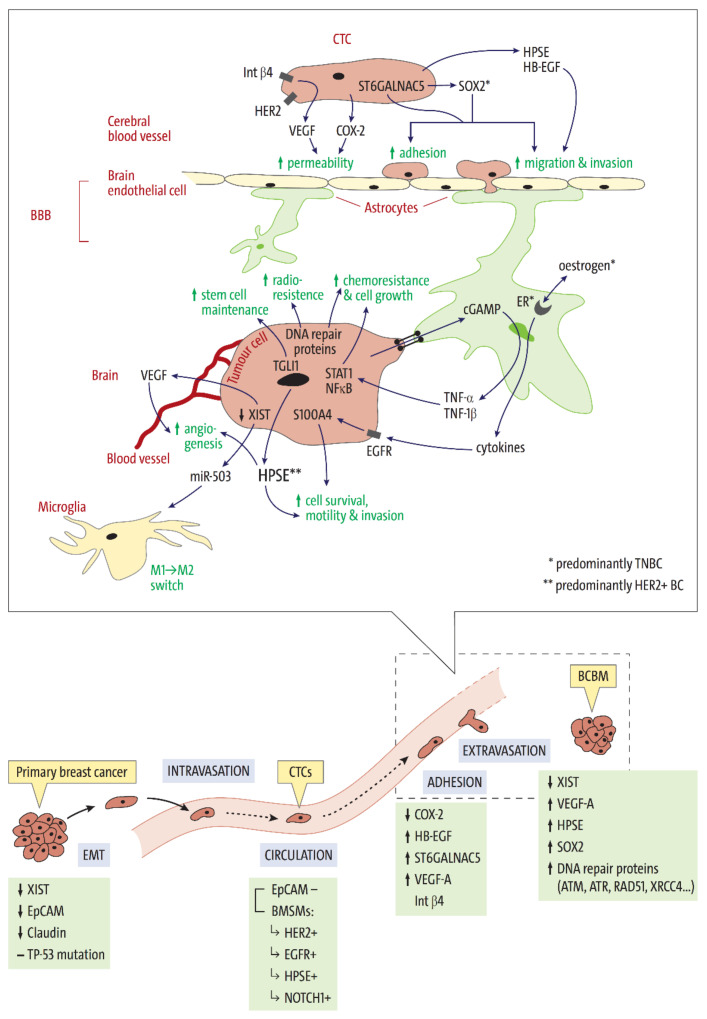
Lower panel: Schematic summary of the stages of breast cancer brain metastases (BCBM) development and the genetic and molecular variables relating to each metastasis step. A breast cancer cell separates from the primary tumor mass via epithelial-mesenchymal transition (EMT) and can enter the bloodstream via intravasation. The now circulating tumor cell (CTC) is transported to blood vessels in distant organs. CTCs which can adhere to vessel walls in the brain and extravasate through the blood-brain barrier (BBB) may succeed in colonizing the brain and proliferating to form a brain metastasis. The downregulation (↓) or upregulation/expression (↑) of each of the listed variables is thought to enable or promote the respective metastasis stage. Zoom box: Factors directly affecting the BBB and a CTC’s ability to metastasize the brain. The expression of VEGF, COX2, and SOX2 in CTCs enhances their ability to adhere to and/or pass through the brain endothelial cell layer via transendothelial migration. Once the tumor cells have transpired the BBB, their interactions within the brain microenvironment are vital for survival. Carcinoma- astrocyte gap junctions enable tumor cells to communicate with neighboring astrocytes, leading to the production of inflammatory cytokines such as TNF-α and TNF-β. Their downstream signaling via the EGFR-, NF-κB- and STAT1 pathways supports tumor cell survival, motility, invasion, and/or chemoresistance. Stimulation of the local microglia causes an M1 to M2 switch, further promoting BCBM growth. Tumor cell-derived VEGF promotes vascular growth and ensures vital blood flow to the growing metastasis. Abbreviations: BC, breast cancer; BCBM, BC brain metastases; XIST, X-inactive-specific transcript; miR-503, exosomal micro-RNA-503; HER2+, human epidermal growth factor receptor 2 enriched/positive; EMT, Epithelial-mesenchymal transition; STAT1/3, Signal transducer and activator of transcription 1/3; NF-κB, Nuclear factor ‘kappa-light-chain-enhancer’ of activated B-cells; BMSMs, Brain metastases selected markers; EGFR, Epidermal growth factor receptor; TNBC, Triple negative BC; EpCAM, Epithelial cell adhesion molecule; CTC, Circulating tumor cell; MAPK, mitogen-activated protein kinase; PI3K, Phosphoinositide 3-kinase; HPSE, Heparanase; NOTCH1, Notch homolog 1, translocation-associated; COX-2, Cyclooxygenase 2; HB-EGF, Heparin-binding epidermal growth factor; ST6GALNAC5, Alpha 2,6, sialyltransferase; ER, estrogen receptor; ITGB4, Integrin Subunit Beta 4 gene; VEGF, Vascular endothelial growth factor; TNF-α/-1β, Tumor necrosis factors-α/-1β; S100A4, S100 calcium-binding protein A4; SOX2, SRY (Sex Determining Region Y)-Box 2; TGLI1, truncated glioma-associated oncogene homolog 1; CNS, central nervous system; exp., expression levels.

Metastasis is a multi-step process in which individual tumor cells, or clusters thereof, disseminate from the primary tumor mass, travel to, and invade a distant organ (Figure 1). Once the tumor cells have invaded the new tissue, they must be able to adapt to the new environment to form a viable secondary tumor. Only a small number of disseminated BC cells survive the metastatic journey and simultaneously possess the genetic profile required to survive as a secondary tumor in the brain [24,25,26]. Several genes have been found to play important roles in a tumor cell’s ability to metastasize (Figure 1 and Table 2). Looking at subtype-associated gene expression may help in understanding how BC subtype might influence BCBM formation.

### 2.1. Tissue Invasion, Intravasation, and Circulation

Firstly, primary tumor cells must separate from the basement membrane to invade local tissues. Epithelial-mesenchymal transition (EMT) describes the process in which the cells undergo the phenotypic changes required to separate from their tumor conglomerate. It is believed that the downregulation of epithelial cell markers such as epithelial cell adhesion molecule (EpCAM), claudin, and E-cadherin, as part of EMT, is an important factor enabling CTCs to extravasate and form metastases [19,27].

Xing et al. performed RNA-expression analyses on BCBM tissues and found that X-inactive-specific transcript (XIST) was significantly downregulated in metastatic brain tissue. XIST is a long non-coding RNA (lncRNA) gene which plays a role in silencing X chromosome-linked genes. Mouse model experiments were able to show that XIST-downregulation in primary BC cells promotes EMT via the activation of the tyrosine kinase c-Met [25]. The downstream pathways of c-Met are associated with proliferation, motility, migration, and invasion [41]. Interestingly, TCGA-database analyses show that 48% of basal-like and 28% of HER2-positive BCs have a decreased XIST expression compared to 19% of luminal A cancers. Furthermore, XIST-downregulation was observed in 78% of primary tumors from women with BCBM, compared to 32% of bone and 41% of lung metastasis patients [25]. Summarized, these results suggest that XIST-expression suppresses the EMT-phenotype and therefore reduces metastasis.

Circulating tumor cells (CTCs) are essential for the development of metastases. Analyses of CTCs from BC patients with BCBM are important in order to understand which CTC-subpopulations possess increased metastatic potential. Characterization of CTCs is, however, difficult, as we lack the technology to unanimously bind all CTCs from a blood sample. This is due to the high phenotypical variability of CTCs, meaning that no one target is expressed on all CTCs. For example, the CELLSEARCH^®^ system uses antibodies to target CTCs expressing EpCAM; however, it does not detect EpCAM negative CTCs. CTCs from BC patients with BCBM frequently lack EpCAM expression. Riebensahm et al. detected CTCs in 47.7% and 32.6% BCBM patients (*n* = 57) after EpCAM-dependent and EpCAM-independent enrichment, respectively [51]. Zhang et al. characterized EpCAM negative CTCs isolated from primary BC patients and were able to identify a potential BCBM molecular signature, which comprises of four “brain metastases selected markers” (BMSMs); HER2+/EGFR+/HPSE+/NOTCH1+ BMSM+ EpCAM negative CTC lines were highly invasive and, when xenografted in mice, led to the formation of brain and lung metastases [27]. Histological analyses also detected the BMSM CTC signature in the metastatic lesions [28].

The epidermal growth factor receptor (EGFR) family is a pivotal focal point for research into the regulation of cancer progression. Two of the four structurally related receptors belonging to this receptor family (EGFR and HER2) have been shown to be highly expressed and/or activated in BC tissue. It is well known that the EGFR-pathway is a frequently dysregulated system in human cancers, aiding tumor growth, progression, and drug resistance [28]. EGFR overexpressing breast carcinoma makes up 15–30% of all primary BCs and is associated with poor patient outcomes [52]. This observation may, in part, may be due the fact that EGFR promotes BCBM development. TNBC’s which express EGFR are more likely to metastasize to the brain than those lacking EGFR [29,30].

HER2, a transmembrane tyrosine kinase encoded by the HER2 gene, is overexpressed in 15% of breast carcinoma. The HER2 signaling cascade activates several pathways, including that of STAT3, Ras-MAPK, and PI3K, which culminate in the inactivation of apoptotic genes and the upregulation of tumor cell proliferation genes [29]. Altered or enhanced HER2 signaling is also associated with an increased risk of metastases formation, and hence reduced patient survival [31,52]. Palmieri et al. used an experimental metastasis model to show that high HER2 expression causes a 3-fold increase in the number of large BM [11]. Hohensee et al. performed genome-wide mutation profiling on primary BC and BCBM tissue and were able to show that aberrations in the EGFR and HER2 signaling pathways were significantly more common in BCBM tissue than that of primary tumors or other distant metastases. In general, material from patients with BCBM was more likely to possess EGFR and HER2 alterations than that from patients without BCBM. Furthermore, EGFR aberrations were most often associated with primary and metastatic tissue of the TNBC subtype and rarely with that of HER2-positive [32]. The strong association between TNBC and EGFR/HER2 mutations helps to explain why this subtype is comparatively prone to develop BCBMs. Furthermore, when considering the findings of Zhang et al. and Hohensee et al., one can hypothesize that CTCs which already possess EGFR/HER2 alterations post intravasation are more likely to invade the brain [27,32].

The NOTCH1 gene encodes a transmembrane receptor which helps control cell specification and differentiation and mediates intercellular interactions between adjacent cells. Activation of the Notch signaling pathway is known to promote primary BC tumorigenesis and is associated with reduced overall survival in primary BC patients [33]. NOTCH1 expression also appears to play a role in BCBM development, as its inhibition results in the reduced formation of BM in vivo [34,35]. Several studies have shown that Notch signaling aids stem cell maintenance [36,37] and, interestingly, that NOTCH1 expression may also contribute to the maintenance of the stem cell phenotype in CTCs [38,39,40]. Stoeck et al. used next-generation sequencing to identify NOTCH1 mutations in a large cohort of tumors [42]. Mutations leading to Notch1 receptor enhancement were singularly found in BCs of the triple-negative subtype [42]. Furthermore, experiments performed by Yuan et al. found Notch activity to be significantly higher in basal-like/triple-negative tissues [43]. In summary, NOTCH1 expression may aid the survival of CTCs originating from TNBC-tissue and enhance their propensity to invade the brain by promoting the stem cell-like phenotype.

### 2.2. Extravasation—Overcoming the Blood-Brain Barrier

The BBB acts as a protective barrier against the invasion of cells, proteins, and chemicals into the CNS. Composed of a tight endothelial cell layer and fortified through the foot processes of neighboring astrocytes, paracellular transport across the BBB is not physiologically possible. Overcoming the high selectivity and adaptive efflux mechanisms of the BBB is a significant hurdle in the development of CNS-affecting anti-cancer drugs [44]. Understanding how circulating BC cells manage to overcome the BBB is an invaluable step in developing therapies which either prevent BCBM formation or eradicate existing metastases.

Three genes have been identified by Bos et al. as mediators of tumor cell passage through the BBB; cyclooxygenase 2 (COX2), heparin-binding EGF-like growth factor (HB-EGF), and alpha 2,6, sialyltransferase (ST6GALNAC5) [9,26]. Prostaglandins such as COX-2 are known to increase the permeability of the BBB during inflammation. In both cell culture and in vitro BBB-model experiments, Bos et al. showed that COX2 knockdown significantly reduced the metastatic activity of brain metastasis-prone cell lines. The HB-EGF gene encodes for the EGFR-ligand HB-EGF, which is highly expressed in many human cancers and is thought to promote tumor cell motility and invasiveness [45]. Primary BC cells expressing COX-2 and HB-EGF undergo enhanced extravasation to both the brain and lungs, while the upregulation of ST6GALNAC5 specifically increases adhesion to brain endothelial cells and thus extravasation in cerebral tissue [26,53]. These genes could be targeted to reduce the extravasation of BC cells into the brain.

The findings of Fan et al. present β4 integrin as an interesting link between HER2-positive subtype and cerebral endothelium cell adhesion and colonization [54]. The β4 integrin subunit fulfills both cell adhesive and signaling functions [55,56]. For example, β4 integrin interacts with HER2, culminating in the production of vascular endothelial growth factor (VEGF). VEGF reduces the integrity of endothelial tight and adherence junctions, enabling adhesion and passage of tumor cells through the BBB. VEGF also helps to promote vascular growth once the tumor cells invade the brain [56]. In summary, these findings suggest HER2-positive tumors are more likely to undergo extravasation because their high level of HER2 receptors mean they are more likely to bind to β4 integrin, which in turn helps them to overcome the BBB.

### 2.3. Intracerebral Metastatic Colonisation

Once tumor cells have successfully overcome the BBB, in order to persevere, they must communicate with and modify the local microenvironment. Perhaps the most important hurdle is evading microglia, the innate immune cells of the brain. When activated, microglia release cytokines and inflammatory molecules with the intention of combating threats to the CNS, such as infection or cancer. Importantly, active microglia can have either a tumor-suppressive (M1) or tumor-promoting (M2) effect, depending on how they are activated. Metastatic tumor cells appear to combat microglial defense mechanisms on two levels: firstly, by directly avoiding the cytotoxic effects of M1 microglia, and secondly, by inducing an M1 to M2 phenotype switch [57].

Returning to the findings of Xing et al., XIST-downregulation also appears to influence the microglial phenotype, aiding cerebral colonization. XIST-downregulation increases the secretion of exosomal micro-RNA-503 (miR-503). Micro-RNA expression, which is often dysregulated in tumor cells, is reflected in exosomal miRNA profiles and can be estimated by measuring ex-miRNA levels in cancer patient blood samples [57]. Secretion of miR-503 alters the STAT3 and NF-κB pathways, culminating in the reprogramming of the microglia phenotype from M1 to M2. In accordance with this finding, Xing et al. observed a positive correlation between miR-503 levels and the development of BM. The authors, therefore, highlight the potential use of miR-503 as a blood-borne biomarker for increased brain metastasis risk [25].

Tumor cells can also stimulate astrocytes in their direct vicinity to increase the production of inflammatory cytokines, such as tumor necrosis factors -α (TNF-α) and -1β (TNF-1β). This is achieved through the formation of carcinoma-astrocyte gap junctions, through which the tumor cells can subsequently transfer the second messenger cGAMP to their neighboring astrocytes. TNF-α and TNF-1β are important inducers of the STAT1 and NF-κB pathways, both of which promote growth and chemoresistance in the newly formed metastasis [24]. Cytokines which upregulate EGFR signaling, and therefore promote tumor colonization have also been found to be produced by ER-expressing astrocytes following hormonal stimulation in a TNBC model. This effect culminates in the expression of S100 calcium-binding protein A4 (S100A4), which promotes cell survival, motility, and invasion [46]. Thus, high circulating estrogen levels of young premenstrual patients may help to explain the fact that young age is an independent risk factor in TNBC for BM development.

Cancer stem-like cells (CSC) are cells which possess stem cell characteristics and make up a small subset of tumor tissue. They can undergo unlimited self-renewal and are highly mobile and invasive [47]. Indeed, it has been proposed that this cell subpopulation is the driving force of metastases development [48]. Several genes relating to the stem cell pluripotency pathway have been found to be amplified in BC metastatic tissue. One such gene is SOX2, which encodes a transcription factor involved in stem-cell and CSC maintenance, plasticity, and survival in the CNS [15,49]. Xiao et al. have shown that increased SOX2 expression in BC cells increases endothelial cell adhesion, transendothelial migration, and in vitro migration across the BBB. High SOX2 expression is significantly associated with reduced BCBM-free survival [50]. Interestingly, increased SOX2 expression is most commonly associated with the TNBC subtype [58]. In accordance with this, of all molecular subtypes, TNBCs exhibit the most CSC-like traits [48,59].

Sirkisoon et al. used mice models to investigate the influence of truncated glioma-associated oncogene homolog 1 (TGLI1) on BCBM formation [60]. TGLI1 is a transcription factor involved in angiogenesis, cell migration, and invasion, which is most highly expressed in HER2-positive and TNBCs; however, its involvement in BCBM formation was yet to be investigated [61,62,63]. Firstly, their results showed that TGLI1 expression leads to increased metastases to the brain and, as such, a reduced BM-free survival, with TGLI1 levels in BCBM tissue being significantly higher than that of the primary BC. Secondly, radioresistant BCBM cell lines and CSCs also showed high TGLI1 expression. TGLI1 appears to be the transcription factor responsible for activating several genes responsible for CSC renewal, including the aforementioned SOX2 gene. Furthermore, TGLI1-positive CSCs subsequently aid astrocyte activation in the BCBM microenvironment. In summary, these findings propose TGLI1 as a link between the primary BC subtypes HER2-positive and TNBC and increased CSC survival and colonization in the brain, culminating in increased BCBM formation [60].

A further factor found to enable cellular movement is the expression of Heparanase (HPSE), an endoglycosidase which cleaves heparan sulfate proteoglycans within the extracellular matrix and basement membrane. As a downstream target of EGFR/HER2 signaling, HPSE is known for its tumorigenic, angiogenic, and prometastatic properties [53,64]. As described above, HPSE is upregulated in CTCs, which show a high propensity to metastasize into the brain, irrespective of molecular subtype [27], and is also found to be overexpressed in BCBM tissue [65,66]. Brain endothelial and glial cells produce HPSE, promoting tumor cell colonization of brain tissue [67].

### 2.4. DNA Repair Mechanisms in BCBM—Survival in the Brain Microenvironment

The brain microenvironment presents a challenging situation for disseminated tumor cells due to elevated levels of endogenous reactive oxygen species (ROS). ROS can induce DNA double-strand breaks, either directly or indirectly, through faults in DNA repair at the replication fork after single-strand lesions. Tumor cells in BM, therefore, require adaptive and optimized DNA-damage defense mechanisms. Such repair mechanisms are indeed one of the most important requirements for the successful establishment of disseminated tumor cells in the brain and are considered a source of therapy resistance.

Increased mRNA expression of DNA repair proteins was observed in BM compared to HER2-positive and TNBC primary BC for 25 of 44 DNA repair proteins examined by Woditschka et al., particularly those associated with homologous recombination (HR) [68]. The XRCC4 protein of the non-homologous end-joining repair pathway, the ERCC1 protein of base excision repair, and the TOPO1 gene associated with repair at replication forks also showed increased expression in BM compared to primary BCs [69,70]. A recent review article identified 268 mutated genes in BM involved in breast cancer-related signaling pathways, regulation of gene transcription, the cell cycle, and DNA repair [71]. Genetic alterations in both activators of the DNA damage response—ATR (Ataxia telangiectasia-related) [72] and ATM (Ataxia telangiectasia-mutated) [73]—were more frequently detected in BM compared with primary BCs and metastases from other sites, respectively. In addition, ATM has been identified as a novel modulator of HER2 protein stability, preventing HER2 degradation and consequently maintaining AKT activation downstream of HER2 [74]. The most common genetic alterations have also been attributed to the DNA repair pathway of HR and HR-regulated signaling pathways in BM. Genes affected by genetic alterations included BRCA1, BRCA2, CHEK2, PALB2, and STAG2 [75,76]. Interestingly, the HR deficiency (HRD) score was higher in BM samples than in primary BC samples [76,77]. The consistent observation that BCBM has higher HRD scores may suggest that they are more sensitive to treatment with PARP inhibitors. Clinical trials should, therefore, adjust their strategy to prioritize patients for PARP inhibitor therapy based on their HRD score.

## 3. Potential Additional Molecular Alterations Associated with Brain Metastasis Development

The genetic complexity of human BC has been unveiled by significant advancements in gene expression analyses over the last two decades, most notably microarray and next-generation sequencing technologies [53]. These methods can be used to analyze large cohorts of cancer tissue samples, leading to the discovery of genetic and molecular variants. Despite the still largely unknown influence of said molecular variants specifically on BCBM development, several potential markers of BM have been proposed.

### 3.1. TP53

The gene TP53 encodes the transcription factor and tumor suppressor protein p53, which plays an important regulatory function in the cell cycle, apoptosis, and DNA repair. Mutations in TP53 which eliminate the protein’s tumor suppressor function are well documented in a variety of human cancers and have been observed in up to 60% of BCBMs [78]. Furthermore, the CNS-prone basal-like and HER2-positive subtypes show significantly higher TP53 mutation rates than the less CNS-prone luminal A and B subtypes. Kobolt et al. found TP53 mutations in 80% of basal-like and 72% HER2-positive primary tumors (compared with 12% of luminal A and 29% of luminal B tumors) [79]. Likewise, 83% of basal-like and 70% of HER2-positive tumors analyzed by Langerød et al. possessed TP53 mutations [80]. The contribution of TP53 mutations to BCBM formation is poorly understood. An important question yet to be answered is whether the basal-like and HER2-positive propensity for BM is associated directly with the TP53 mutation or whether both observations (TP53 mutation and metastatic tendency) are independently associated with a molecular subtype.

### 3.2. PI3K/Akt/mTOR-Pathway

Phosphoinositide 3-kinase (PI3K) is a transmembrane protein with a catalytic subunit responsible for phosphorylating phosphatidylinositol-4,5-bisphosphate (PIP2) to create PIP3. PIP3 is then able to recruit, phosphorylate and activate proteins such as Akt, which initiate several downstream effects within the cytoplasm [81,82]. As such, the PI3K/Akt pathway mediates a variety of cellular functions, which are fundamental for tumor initiation, growth, motility, angiogenesis, and survival [83]. Mutations which cause upregulation of this pathway, for example, in the PIK3CA gene, are observed in a variety of cancer entities and are significantly associated with decreased patient overall survival. Approximately 50% of primary BCs exhibit PI3K/Akt pathway upregulation [81,84]. The PI3K/Akt pathway is also upregulated in most BCBMs, irrespective of molecular subtype [81]. The significance of this pathway specifically in the development of BCBM is, however, yet to be fully understood.

### 3.3. PTEN

The PI3K/Akt pathway is negatively regulated by the lipid phosphatase PTEN (Phosphatase and Tensin Homolog). In counteraction to PI3K, PTEN dephosphorylates PIP2 and PIP3 and therefore inactivates the downstream cascade [81]. PTEN loss-of-function mutations, therefore, result in PI3K/Akt pathway activation. PTEN loss rarely occurs in primary BC but is often observed in BCBM tissue [85]. Mutation profiling conducted by Hohensee et al. revealed that 21% of BCBM possessed PTEN mutations [32]. In functional studies, upregulation of PTEN in a TNBC cell line led to reduced migration and invasion to the brain. Here, the crosstalk between tumor and glial cells, mediated by activation of GM-CSF/CSF2RA and AKT/PTEN pathways on both astrocytes and tumor cells, was demonstrated to play a key role [86]. Gonzalez-Angulo et al. performed immunohistochemical and mass spectroscopy analyses on BC and BCBM matched pairs and observed a 26% PTEN discordance between matched pairs. However, this included five cases of PTEN loss and eight cases of PTEN gain following brain colonization, which suggests that conversion is a stochastic event and, therefore, that metastatic competence is not dependent on this mutation [85]. Further research must be conducted to clarify the importance of PTEN loss or gain in BCBM formation.

### 3.4. Low Methylation Levels in TNBC

Although all BCBM have lower methylation levels compared to their primary BCs, the TNBC subtype has been associated with the comparatively lowest methylation levels of all molecular subtypes [14,16]. This finding is noteworthy, as hypomethylation has been associated with metastatic invasiveness in other cancer forms, such as colorectal cancer [87]. This is an interesting finding which remains to be further investigated.

### 3.5. Metabolic Phenotype

Of all organs, the brain has the highest glucose demand and therefore favors enhanced glycolytic activity for energy production [88]. Accordingly, increased glycolysis has been observed in BCBM when compared with bone or lung metastatic tissue [89]. Interestingly, the metabolic phenotype also varies between the different molecular BC subtypes. In contrast to the comparatively metabolically inactive luminal subtypes—which show a reverse-Warburg/null phenotype -, TNBC displays a Warburg/mixed phenotype, characterized by high glycolysis and low mitochondrial respiration [90,91]. HER2-positive tumors also generally exhibit a glycolytic phenotype [92,93]. Here, Ding et al. described that HER2 translocation to the mitochondria, induced by its interaction with the heat shock protein-70 (mtHSP70), negatively controls oxygen consumption, consequently enhancing glycolysis [94]. Interestingly, some of the aforementioned genetic alterations have been described to regulate the metabolic activity in tumor cells. For example, TP53 mutations have been shown to increase glycolytic activity by activating the RhoA/ROCK/GLUT1 signaling cascade [95]. Furthermore, the activation of the PI3K/Akt/mTOR pathway enhances the expression of genes related to glucose uptake and glycolysis through normoxic upregulation of HIF-1a [96,97]. However, the metabolic phenotype may not only be determined by the molecular subtype but also dependent on the interplay between cancer cells and their microenvironment [98]. Thus, metabolic plasticity is an important characteristic distinguishing tumor cells with high metastatic potential from non-metastatic tumor cells.

## 4. Clinical Implications and Perspectives: How Is Our Current Understanding of BC Subtype and Its Influence on BCBM Formation Being Used to Combat BC Brain Metastasis?

Knowledge of a tumor’s molecular subtype not only enables assessment of BM risk but is also an important determinant of metastases therapy effectiveness. The molecular subtype of a patient’s primary and metastatic tumors should therefore be taken into consideration when choosing the appropriate therapy [10]. The findings presented thus far are the basis for several pharmacological research projects aiming to develop specific therapies to combat BCBM based on a tumor’s molecular footprint. Although a portion of this research remains at an experimental level, with only a small number of trials having reached a clinical phase for BCBM patients, these developments highlight the important connection between knowledge at a genetic and molecular level and the eventual development of effective, personalized therapies.

### 4.1. XIST as a Therapy Target

Xing et al. used mouse models to further validate their finding that low XIST expression (XISTlow) enhances BCBM formation. In doing so, they simultaneously proposed a potential therapy for this patient collective. They treated low XISTlow BC mouse models with fludarabine, a synthetic drug which is selectively lethal for XISTlow BC cells. Mice that received the treatment and were, therefore, rid of all tumor cells with reduced XIST expression showed significantly delayed BCBM onset and a significantly slower BCBM growth rate [25]. This is an interesting and promising result. Fludarabine can cross the BBB, shows low neurotoxicity, and has already been approved for human usage in cases of leukemia, rendering it a potential therapeutic agent for metastatic BC patients with XISTlow tumor cells [99].

### 4.2. Anti-HER2 Treatment in BCBM Patients

The anti-HER2 monoclonal antibody trastuzumab is a successful therapeutic agent for HER2-positive primary BC and, logically, is being tested for its efficacy against HER2-positive BCBM [100]. Indeed, the onset of symptomatic BM can be delayed through treatment with trastuzumab; however, the utility of the drug as a systemically applied therapy is restricted, as it does not cross the BBB in sufficient concentrations needed to elicit these anti-BCBM effects [101]. Several techniques are currently being tested to combat this problem, such as intrathecal application of the antibodies, increasing the pharmaceutical dosage or administration in combination with other therapeutic agents, as well as radiotherapy to increase the BBB permeability [102,103,104,105,106].

The most remarkable treatment for HER2-positive patients has recently been seen with the small-molecule tyrosine kinase inhibitor (biologics) tucatinib, which has been tested in the HER2CLIMB trial as a combined anti-HER2 therapy for women with HER2-positive BC with or without BM. In patients with stable or active BM, the addition of tucatinib to trastuzumab and capecitabine doubled the intracranial objective response rate (47.3% vs. 20.0%). The estimated 1-year CNS-progression free survival (PFS) was 40.2% (95% CI, 29.5% to 50.6%) in the tucatinib arm and 0% in the control arm, indicating tucatinib showed efficacy in controlling intra- and extracranial disease by prolonging PFS in the overall population of patients with BM [107].

### 4.3. The PI3K/Akt Pathway Is Uniquely Active in BCBM

As described above, mTOR is an important element in the PI3K/Akt signaling cascade, and therefore plays an essential role in angiogenesis, cell growth, proliferation, and survival. This pathway has been shown to be uniquely active in BCBM and is, therefore, an interesting target for novel therapies [108]. Therapy concepts were extended to include everolimus, a selective mammalian target of rapamycin (mTOR) inhibitor.

PI3K targeting is a pharmacological principle which has already proven effective in treating systemic BC metastases [109]. Interestingly, brain metastases, in particular of the HER2-positive subtype, seem to show resistance to PI3K inhibitors. This is in part be due to the development of HER2-HER3 heterodimers, which strongly activate the PI3k/Akt pathway and counteract the effects of the inhibitors [109,110]. HER3 expression is often augmented in HER2-positive BM, increasing the frequency of HER2-HER3 heterodimers. Kodack et al. found that blocking HER3 in HER2-positive BCBM mice models eliminated the resistance of the metastases to PI3K inhibitors. These mice experienced a significant delay in tumor growth and survived longer [109]. These results highlight the complexity of the brain’s microenvironment and the need to develop brain- and subtype-specific therapies for BCBM.

### 4.4. VEGF Antibodies in Combination with Anti-HER2 Therapy

Another promising combination is that of trastuzumab and lapatinib with bevacizumab, an anti-VEGF antibody. This triple therapy was initially tested on HER2-positive BCBM mouse models, where bevacizumab was found to reduce the extravasation of CTCs and inhibit metastatic vascular growth [13,111]. Subsequent clinical trials support these findings and show that the cytotoxicity of the anti-HER2 agents is directly enhanced through their combination with bevacizumab. It is hypothesized that therapeutic tumor penetration is optimized by this drug combination [112].

### 4.5. Oestrogen Depletion as Potential BM Prevention in TNBC

The findings of Sartorius et al. suggest that anti-estrogen therapies could be implemented as TNBC therapy to prevent the development of BM [46]. As described above, estrogen has been found to endorse ER-positive astrocyte production of cytokines which support migration and proliferation of metastatic cells. Research on preclinical TNBC models shows that estrogen depletion significantly reduces BM development [46]. These findings imply that ovarian estrogen depletion or therapy with aromatase inhibitors may be an effective form of BM prevention in high-risk cases of TNBC. This hypothesis should be further investigated.

## 5. Limitations and Unanswered Questions

### 5.1. Genetic Alterations during the Metastatic Process and/or Therapy

It is important to note that the molecular subtype of BM does not always correlate with that of the primary tumor [113]. Cejalvo et al. compared the intrinsic subtypes and gene expression in 123 paired primary and metastatic tissues, showing that subtype conversion occurred in 55.3% of luminal A, 30% of luminal B, 23,1% of HER2-positive, and 0% of TNBC; 14.3% of luminal A and B tumors converted to HER2-positive [114]. Furthermore, 47 genes were found to be differentially expressed in primary versus metastatic tumors [114]. A retrospective study conducted by Sperduto et al. found a subtype-altering receptor discordance between primary BC and BCBM samples in 32% of BCBM patients. Among these, 13% of HER2-negative BC gained de novo HER2-positivity after metastasis [115]. Zhang et al. observed that CTCs derived from a TNBC (ER-/PR-/HER2low) patient with BM were positive for EGFR and HER2 at an mRNA and protein level [27]. Similarly, Hohensee et al. found that the HER2-positive status was more common in BCBM than primary BC samples, with 17% of matched pairs showing de novo HER2-positivity [32].

How do metastatic tumor cells come to have a different subtype to those from their tumor of origin? Firstly, cases of multiclonal seeding from primary tumors composed of different cell populations have been observed. This is thought to be a common occurrence in TNBC, as most tumors of this subtype are polyclonal, as described above [2,116]. Secondly, exposure to therapeutics might also alter gene expression and, therefore, BC molecular subtype. Recently, Kim et al. used single-cell DNA and RNA sequencing analysis to show that chemoresistant TNBC cells pre-existed in the primary, chemo-naïve tumor and underwent an adaptive selection during neoadjuvant therapy [117]. While the genotype of these cells remained unaltered, the transcriptional profile was reprogrammed in response to the therapy [117]. Thirdly, the vascular or cerebral microenvironment can also impact gene expression and the molecular subtype of tumor cells once they have left the primary tumor or metastasized to the brain [19,118].

The plasticity of tumor cell molecular identity is undeniable. Nonetheless, there does seem to be an overall high degree of similarity between gene expression and molecular subtype in primary BC and metastases matched pairs [119]. Harrell et al. found that over 90% of 298 tested genes remained stable following metastases, showing that the similarities between matched pair gene expression strongly outweigh the differences [19]. This suggests that primary tumors are good predictors of metastatic propensity.

Despite these findings, we do not fully understand the mechanisms of subtype conversion after metastases formation, nor do we completely understand the significance of this conversion when it does take place. The findings do, however, suggest the need for personalized and BM-specific therapies. Studies looking into the role of molecular discordance on prognosis present inconsistent data [120]. Understanding when and why subtype conversion takes place may help to discern which special precautions are warranted when treating this patient collective.

### 5.2. Difficulties in BCBM Therapy Development

Systemically applied chemotherapeutic agents are limited in their efficacy when treating intracranial metastases [121]. As previously mentioned, the impassability of the BBB and its specialized and adaptable transmembrane efflux pumps impede the application of an effective drug concentration in the vicinity of the metastases. It has also been suggested that abnormal local perfusion in the metastasis’s vicinity can further impede drug delivery [122]. These difficulties may be overcome with the implantation of strategically placed intracerebral or intrathecal catheters, as is currently being tested with trastuzumab [102].

## 6. Conclusions

Although the prognosis of metastatic BC with BM remains poor, scientists and clinicians are rapidly gaining knowledge on the molecular intricacies of the disease. Several possible genetic and molecular mechanisms behind the propensity of HER2-positive and TNBC tumors to metastasize to the brain have been presented in this review article. Firstly, TNBC and HER2-positive tumors are most likely to exhibit alterations in the EGFR- and HER2-associated signaling pathways, which in turn are related to increased development of BM and a poorer prognosis. These mutations have also been found to enhance the formation and survival of circulating tumor cells (CTCs). Secondly, both molecular subtypes are more likely to show XIST downregulation, which promotes epithelial-mesenchymal transition, motility, and migration of primary BC cells and specifically enhances the tendency of CTCs to invade the brain. CTC passage through the BBB and colonization in the brain is furthermore enhanced by the pro-metastatic effects of β4 integrin, to which HER2-positive tumors are most susceptible. Thirdly, of all molecular subtypes, basal-like TNBC most often contains cancer stem-like cells (CSCs). SOX2, the transcription factor responsible for maintaining the CSC phenotype, is also most often highly expressed in the TNBC subtype. This highly mobile and invasive cell subpopulation significantly enhances the BM potential of primary BC. Several further molecular traits have been linked to increased BM formation, for example, PI3k/Akt pathway activation and loss of TP53 tumor suppressor function, and present important targets for potential anti-BCBM therapies.

As has been shown in this review, the importance of molecular subtype and gene expression on BCBM development is undeniable. This realization has been supported by fundamental research for nearly two decades and is now being reflected in research at preclinical and clinical levels. Importantly, a drift in molecular characteristics can take place under therapeutic exposure or due to a changing microenvironment during the process of metastases. This complicates the development and perhaps reduces the efficacy of BCBM therapies when they are chosen based on the primary BC subtype alone. We are therefore observing the trend toward therapies which are also specialized for the molecular footprint of CTCs and existing metastases. Currently, BCBM is regarded as an incurable complication of primary BC. However, based on our rapidly growing understanding of BC molecular subtypes and BM biology and the consequential development of increasingly targeted therapies, we can hope to see improvements in patient outcomes in years to come.

## Figures and Tables

**Table 1:** Overview of the four clinically relevant molecular subtypes of BC, based on the expression of hormone and growth factor receptors and the proliferation marker Ki-67, and their association with brain metastasis development.

Subtype	Hormone Receptor		Growth Factor Receptor	Proliferation Marker	% of Invasive BC	5-Year-Risk (%) of Brain Metastases after BC Diagnosis	Literature
	ER	PR	HER2	Ki-67			
Luminal A	(+)	(+)	−	low	50%	0.1%	[9,10,11]
Luminal B	(+)	(+)	−	high	20%	~3.3%	
HER2-positive	±	±	+	high	15%	3.2%/3.7% *	
TNBC	−	−	−	high	~15%	7.4%	

Abbreviations: BC, breast cancer; TNBC, triple-negative breast cancer; ER, estrogen receptor; PR, progesterone receptor; HER2, human epidermal growth factor receptor 2; +, positive; −, negative; (+), hormone receptor-positive for ER and/or PR; ±, positive or negative. * Respective 5-year-risk of brain metastases for primary hormone receptor-positive/hormone receptor-negative HER2-positive breast cancers.

**Table 2:** Overview of genes and molecular markers associated with increased BCBM formation, including the associated primary BC subtype and/or tissue type in which they are most commonly detectable and the mechanism by which BM development is thought to be promoted. Shaded rows roughly divide the markers into groups based on the metastases phase in which the variable exerts its influence.

Genetic Marker	Primary BC Subtype	Tissue Type Other than Primary BC	Mechanism	Associated with		Literature
				Increased	Reduced	
**EMT and Circulation (CTCs)**						
**XISTlow**	TNBC (48%), HER2+ (28%), Lum. A (19%)	BCBM	↓ XIST → ↑ c-Met → STAT3 and PI3K pathway activation	tumour cell proliferation, motility, migration, and invasion		[27]
**EGFR**	25–30% of BC, most often TNBC	EpCAM negative CTCs, BCBM	STAT3, Ras-MAPK and PI3K pathway activation	tumour cell proliferation, invasiveness, brain metastases	apoptosis, patient survival	[14,17,28,29,30,31,32]
**HER2**	15–35% of BC					
**NOTCH1**	TNBC		Gene mutation → ↑ Notch1 receptor → ↑ Notch signaling	primary BC tumorigenesis, stem cell and CTC maintenance	overall survival	[33,34,35,36,37,38,39,40]
**Extravasation**						
**COX2**	ER-negative tumour cells	BCBM-cell lines		BBB permeability		[41]
**HBEGF**			↑ EGFR-ligand HB-EGF	tumour cell motility and invasiveness		[41,42,43]
**ST6GALNAC5**				BC-cell adhesion to brain endothelial cells		[41,43]
**ITGB4**	HER2+		β4 integrin interaction with HER2, VEGF production	adhesion and extravasation of BC cells through BBB, vascular growth in BCBM microenvironment	endothelial tight and adherence junctions, VEGF-dependent endothelial integrity	[44]
**Cerebral Colonization**						
**XISTlow**	BLBC (48%), HER2+ (28%), Lum. A (19%)	BCBM	↓ XIST → ↑ miR-503 → ↑ STAT3 and NF-κB signaling → microglia phenotype switch M1 to M2		microglial defence against invading tumour cells	[27]
**S100A4**	TNBC cells	ER-expressing astrocytes following oestrogen stimulation (TNBC model)	Oestrogen stimulation → cytokines → ↑ EGFR-signaling → ↑ S100A4	cell survival, motility and invasion		[45]
**TGLI1**	HER2+ and TNBC (low exp.)	BCBM (high exp.), CSCs, radioresistant BCBM cell lines	transcription factor → activation of several genes incl. SOX2	CSC renewal, astrocyte activation in BCBM microenvironment	BM-free survival	[46]
**HPSE**	HER2+	CTCs (all subtypes), BCBM, brain endothelial and glial cells	EGFR/HER2 signaling → HPSE	tumorigenesis, angiogenesis, and metastasis		[28,42,47,48,49,50]
**SOX2**	TNBC BLBC	BCBM	transcription factor	stem-cell maintenance in the CNS, tumour cell plasticity and endothelial cell adhesion, trans-endothelial migration, and migration across the BBB	BM-free survival	[18,48,49,50,51,52]

Abbreviations: BC, Breast Cancer; EMT, Epithelial-Mesenchymal-Transition; CTC, Circulating Tumor Cells; XIST, X-inactive-specific transcript; TNBC, Triple Negative Breast Cancer; HER2+, Human epidermal growth factor receptor 2 enriched/positive; Lum., Luminal; BCBM, Breast Cancer Brain Metastasis; STAT1/3, Signal transducer and activator of transcription 1/3; EGFR, Epidermal growth factor receptor; EpCAM, Epithelial cell adhesion molecule; NOTCH1, Notch homolog 1, translocation-associated; COX-2, Cyclooxygenase 2; ER, Estrogen Receptor; BBB, Blood-Brain-Barrier; HB-EGF, Heparin-binding epidermal growth factor; ST6GALNAC5, Alpha 2,6, sialyltransferase; VEGF-A, Vascular endothelial growth factor A; BLBC, Basal-Like Breast Cancer; miR-503, exosomal micro-RNA-503; NF-κB, Nuclear factor ‘kappa-light-chain-enhancer’ of activated B-cells; S100A4, S100 calcium-binding protein A4; CNS, Central Nervous System; TGLI1, truncated glioma-associated oncogene homolog 1; HPSE, Heparanase; downregulation (↓) or upregulation/expression (↑).
